# Associations between Fatty Acid Intake and Status, Desaturase Activities, and *FADS* Gene Polymorphism in Centrally Obese Postmenopausal Polish Women

**DOI:** 10.3390/nu10081068

**Published:** 2018-08-10

**Authors:** Agata Muzsik, Joanna Bajerska, Henryk H. Jeleń, Anna Gaca, Agata Chmurzynska

**Affiliations:** 1 Institute of Human Nutrition and Dietetics, Poznan University of Life Sciences, 60-624 Poznan, Poland; agata.muzsik@up.poznan.pl (A.M.); joanna.bajerska@up.poznan.pl (J.B.); 2 Institute of Food Technology of Plant Origin, Poznan University of Life Sciences, 60-637 Poznan, Poland; henryk.jelen@up.poznan.pl (H.H.J.); anna.gaca@up.poznan.pl (A.G.)

**Keywords:** fatty acids, dietary intake, fatty acid desaturase (*FADS*) gene, postmenopausal women

## Abstract

Fatty acid (FA) status is associated with the risk of several diet-related diseases. Since postmenopausal women are at increased risk of cardiometabolic disturbances, determinants of FA metabolism should be fully understood in this group. We hypothesize that FA metabolism in postmenopausal Polish women may depend on current macronutrient intake and on fatty acid desaturase (*FADS*) gene polymorphism. One-hundred-and-twenty-eight postmenopausal women with central obesity were recruited to the study and their dietary intake, FA composition in red blood cells (RBC), and rs174556, rs174561, rs174547, and rs3834458 polymorphism of the *FADS* gene were analyzed. Higher levels of 18:2n-6t level in RBC were associated with higher protein or fat intake or with lower carbohydrate intake. The minor allele carriers of rs174561 of the fatty acid desaturase 1 (*FADS1*) gene had 9.7% lower concentration of 20:4n–6 in RBC (*p* < 0.05), but there were no other associations between other FA in RBC levels and *FADS1* or fatty acid desaturase 2 (*FADS2*) polymorphisms. The mean D5D value was 15.3–17.9% lower in the minor allele carriers of each SNPs. We concluded that protein and carbohydrate intake may be associated with FA concentrations in RBC in centrally obese postmenopausal Polish women. The D5D value may be affected by *FADS1* or *FADS2* polymorphism.

## 1. Introduction

Fat and fatty acid (FA) composition in diet has a significant effect on health, and thus plays an important role in the development of noncommunicable diseases, such as overweight, obesity, metabolic syndrome (MetS), and nonalcoholic fatty liver disease. Postmenopausal women, who show slower energy metabolism, are more prone to these metabolic disorders, especially disturbances of lipid metabolism [1,2,3,4,5]. Additionally, the postmenopausal diet is characterized by a low intake of vegetables and fiber and a high intake of protein and animal fat, which results in higher intakes of saturated fatty acids (SFA) and lower unsaturated fatty acids (unSFA) [6,7,8,9]. Similarly, in postmenopausal Polish women with dyslipidemia, lower polyunsaturated fatty acids (PUFA) intake and higher SFA intake have also been observed [10].

**Figure 1:**
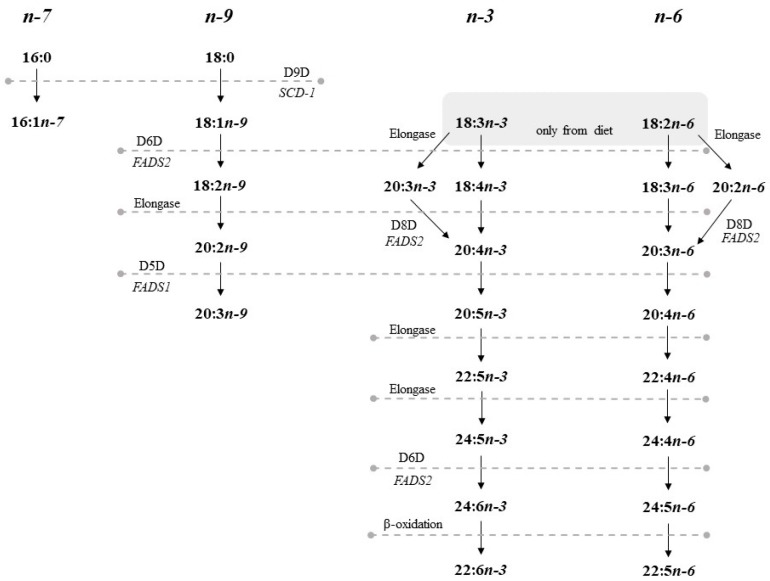
Endogenous pathways of polyunsaturated fatty acids (PUFA) metabolism in mammals. D5D: Delta-5 desaturase; D6D: Delta-6 desaturase; D8D: Delta-8 desaturase; D9D: Delta-9 desaturase; SCD-1: Stearyl-CoA desaturase 1; *FADS1*: Fatty acid desaturase 1; *FADS2*: Fatty acid desaturase 2; PUFA: Polyunsaturated fatty acids.

FA nutritional status and the metabolic health of postmenopausal women depend not only on the dietary intake of FAs, but also on their endogenous synthesis (Figure 1). The metabolism of long-chain polyunsaturated fatty acids (LC-PUFAs) consists of alternate reactions of desaturation and elongation. It also depends on dietary precursors, which mammals cannot produce. α-linolenic acid (ALA, 18:3n-3) is a precursor in the synthesis of eicosapentaenoic acid (EPA, 20:5n-3) and docosahexaenoic acid (DHA, 22:6n-3), while linoleic acid (LA, 18:2n-6) is a precursor in the synthesis of arachidonic acid (AA, 20:4n-6). LC-PUFA pathways are limited by the enzymes 5-Δ-desaturase (D5D) and 6-Δ-desaturase (D6D), which are encoded by fatty acid desaturase 1(*FADS1*) and *2* (*FADS2*) genes, respectively (Figure 1) [11,12,13]. 

The activities of D5D and D6D depend on different factors, including genetic polymorphism, aging, dietary PUFA, metabolic disorders, and blood lipids [12,14,15,16,17,18,19]. Enzyme activity can also be affected by single-nucleotide polymorphisms (SNPs) of *FADS1* and *FADS2*. *FADS* genotypes account for up to 28% of the variability in FA concentrations in serum phospholipids [11]. There have been a number of studies showing associations between variations in *FADS* (rs174556, rs174547, rs174561, and rs3834458) and FA status, and the majority reported that minor allele carriers have decreased synthesis of LC-PUFA with higher proportions of ALA to EPA and DHA and LA to AA [11,12,13,20,21,22,23,24,25,26].

There have been several studies to analyze FA metabolism, FA enzyme activity, or *FADS* gene-cluster polymorphism in adults [11,12,13], but these have focused on patients with MetS or diabetes [27], pregnant or lactating women [28], elderly people [29], or subjects with cardiovascular disease [30]. There have only been a few studies to consider all these factors together [11,12,13,14,20,30,31,32]; these were cross-sectional studies that examined both women and men together, without age subgroups. For this reason, there is still a lack of data on FA status in postmenopausal women. Since FA status is associated with the risk of several diet-related noncommunicable diseases, the determinants of FAs metabolism and their concentrations in blood and tissues should be fully understood in different populations. This is of special importance in postmenopausal women, where lipid metabolism abnormalities are often observed.

We hypothesize that FA metabolism in postmenopausal Polish women depends on current protein, carbohydrate, fat, and FA intake, and on *FADS* gene polymorphism. The primary aim of this study is to investigate the relationship between FA intake or *FADS1* and *FADS2* polymorphism with the LC-PUFA metabolism. A secondary aim is to determine the effect of *FADS* gene polymorphism on the activities of fatty acid enzymes, such as D5D and D6D. To this end, FA intake, FA profile in red blood cells (RBC), and *FADS1* and *FADS2* genotype were examined in a group of postmenopausal Polish women.

## 2. Materials and Methods 

### 2.1. Subjects

The subjects were recruited in 2014 through advertisements in local newspapers [33]. Women in postmenopausal period and under 70 years of age were included for the study. The postmenopausal period was defined as at least 1 year since the last menstrual period plus a follicle-stimulating hormone concentration of 30 IU/L at screening. The exclusion criteria were participation in a weight loss therapy or weight fluctuation in the 6 months prior to the current study, intolerance or food allergies, a history of heart disease, insulin-dependent diabetes or type 2 diabetes, kidney disease, hypothyreosis, chronic inflammatory disease, liver disease, osteoporosis, any type of cancer, and tobacco smoking. Additionally, women were excluded if they were undergoing hormone replacement therapy or medication for a psychiatric disorder. Poznan Medical University approved the protocol for the study (number 603/14). All participants gave their written informed consent before enrollment to the study.

### 2.2. Dietary Assessment 

Dietary intake was assessed using a 3-day food diary in which the participants were clearly instructed to record information on nonconsecutive days (2 weekdays and 1 weekend day) regarding their food, beverage and supplements intake [34], using household measures. The food and beverage quantities thus obtained were converted into grams and milliliters and computed using the dietary analysis software Dieta 5.0 (National Food and Nutrition Institute, Warsaw, Poland); the average intake of nutrients (protein, fat, carbohydrates, FA, cholesterol) and energy was thus calculated. The Polish dietary standards were used to assess adequate intakes [35].

### 2.3. Anthropometry

The anthropometric data included body weight, height, body mass index (BMI = weight/height^2^), and waist circumference. Height was measured to the nearest 0.5 cm by stadiometer WPT 100/200 OW (RadWag, Poznan, Poland). Body weight was measured to the nearest 0.1 kg with subjects in a bathing suit following an overnight fast, using a calibrated scale included in the Bod Pod (Cosmed, Rome, Italy). Waist circumference was measured at the midpoint between the lowest rib and the top of the iliac crest using nonelastic tape. This measurement was performed by a single evaluator. Women with waist circumference ≥ 80 cm were considered centrally obese [36]. 

### 2.4. Physical Activity

Physical activity level was assessed using the short version of the International Physical Activity Questionnaire (IPAQ), which has been validated for youths and adults. Moderate and intensive physical activity, work, active commuting, and household activity were calculated from their durations in the previous week [37].

### 2.5. Analysis of Fatty Acid Profile in Erythrocytes

Erythrocytes were separated from whole blood by density gradient centrifugation by centrifuge 5702R (Eppendorf, Hamburg, Germany), washed with phosphate buffer saline (PBS), and stored at −80 °C. FAs were extracted using the modified Folch method [38]—briefly, the Folch reagent (2:1 chloroform: methanol) for extraction of lipids from cells, butylated hydroxytoluene as an antioxidant and internal standard: deuterated myristic-d_27_ acid (d_27_C14:0) in chloroform were added to weighted samples and the mixture was centrifuged. The approximate 2 mL of the lipid-containing chloroform phase was removed and derivatized with 0.5 mL of 0.5M potassium hydroxide in methanol and 1 mL of boron trifluoride methylation by heating at 70 °C. Fatty acid methyl esters (FAME) were extracted into 4 mL of hexane and washed out with 2 mL of distilled water. The organic (hexane) phase was then transferred into 2 mL vials and subsequently analyzed by gas chromatography. The analysis was performed on a Hewlett-Packard 6890 gas chromatograph (Wilmington, DE, USA) equipped with a split–splitless injector and a flame ionization (FID) detector. FAME were separated using a SelectFame column (50 m × 0.25 mm × 0.25 μm, Agilent Technologies, Santa Clara, CA, USA identified by comparison with available FAME standards (Supelco, Bellefonte, PA, USA). Fatty acid content was calculated by comparing individual peak areas with the peak area of an internal standard and recalculated based on sample weight. Response factors (FID) for particular fatty acids versus internal standards were assumed to be unity. The concentration of fatty acids in RBC was expressed as µg/mL.

### 2.6. Desaturase and Elongase Activities

Desaturase and elongase activities were calculated using product-to-precursors ratios. The D5D and D6D were calculated using 20:4n-6/20:3n-6 and 18:3n-6/18:2n-6 ratios, respectively. The combined effects of desaturase and elongase activities ratios were calculated as follows: 20:4n-6/18:2n-6 and 22:4n-6/18:2n-6 in n-6 PUFA metabolism and 20:5n-3/18:3n-3, 22:6n-3/18:3n-3 and 22:6n-3/20:5n-3 in n-3 PUFA metabolism.

### 2.7. Genotyping

The rs174556, rs174547, and rs174561 polymorphisms of the *FADS1* gene and the rs3834458 polymorphism of the *FADS2* gene were selected for analysis. The blood was collected into tubes containing EDTA, and the DNA isolated from fresh blood using a NucleoSpin Blood kit (Macherey–Nagel, Düren, Germany). Genotyping was performed with the use of hybridizing probes designed and synthesized by Tib MolBiol (Tib Molbiol, Berlin, Germany), as shown in Table 1, on a LightCycler 480 instrument (Roche, Basel, Switzerland). One probe was labeled with fluorescein and the other with LC640 dye. PCR was run using 10 µL reaction mix at a primer concentration of 0.5 µM/µL and a probe concentration of 0.15 µM/µL. The reaction was performed in 96-well format in a total reaction volume of 10 µL, using 20 ng of genomic DNA. The cycling profile was 95 °C for 10 min, followed by 45 cycles at 95 °C for 10 s, 61 °C for 10 s, and 72 °C for 15 s. Genotypes were analyzed using a melting curve method.

### 2.8. Statistical Analysis

Student’s *t*-test was applied to compare the crude means. Adjustment of continuous variables was performed by linear regression and the models were adjusted for BMI, physical activity, and whether hypolipidemic or hypoglycemic medications were being taken. The models for desaturase activities were also adjusted for age and intake of enzyme precursors. The group was stratified by nutrient intake with low or high intake here meaning below or above the median values. Intake was considered as % energy from a particular nutrient. Additionally, protein intake was considered as a categorical variable (inadequate or inadequate). Adequate protein intake means ≥1.2 g of protein/kg of body mass/day. *p* < 0.05 was considered statistically significant. Data were analyzed using Statistica software (StatSoft, Tulsa, OK, USA).

## 3. Results

### 3.1. Subjects Characteristics

One-hundred-and-forty-three women provided dietary data and 128 out of them provided blood samples. The mean age was 60.7 ± 5.1 years and the average BMI was 33.7 ± 4.9 kg/m^2^. All women had central obesity and the mean waist circumference was 105.2 ± 9.6 cm. 

### 3.2. Dietary Intake of Macronutrients and FA

The characteristics of the macronutrient and FA intakes are described in Table 2. The mean total energy intake was 1699 ± 59.5 kcal, which did not include energy from dietary fiber. The mean percent energy intakes from protein, fat, and carbohydrates were 17.1 ± 0.4%, 28.2 ± 0.7%, and 53.9 ± 0.9%, respectively. The mean percent energy intake from SFA was 10.9%, while those from monounsaturated fatty acids (MUFA) and PUFA were 11.8% and 4.1%, respectively. The mean PUFA+MUFA/SFA ratio was 1.5 ± 0.04, and more than 93.0% of participants had an excess intake of the percent energy from SFA.

### 3.3. Associations between Macronutrient Intake and FA Composition In Erythrocyte Membranes

We examined how macronutrient intake affected FA composition in erythrocyte membranes. Higher protein intake was associated with higher concentrations in RBC of 16:1n-7, 18:1n-7c, 18-1n-5c, and 18:2n-6t (*p* = 0.035, 0.022, 0.007, and 0.001, respectively; see Table 3). The opposite effect was observed for 17:1, 18:1n-7t, 22:1n-9, 22:4n-6 and 22:6n-3 in RBC. A lower intake of carbohydrates (below the median value) was significantly associated with the 21.5% higher level of 18:2n-6t (*p* < 0.05) and the 19.9% lower level of 17:1 (*p* < 0.05; Table 3). Higher percentage energy intake from fat (above the median value) was associated with 26.0% higher concentration of 18:2n-6t (*p* < 0.05) and 9.0% lower concentration of 22:4n-6 (*p* < 0.05; Table 3). Moreover, no associations were seen between fat intake and other FA concentrations in erythrocyte membranes. We also examined the associations between percentage energy from SFA, PUFA, MUFA, and FA concentrations in RBC, and no general associations were found (Supplementary Materials, Tables S1–S3). The only significant result was an association between higher percentage energy from MUFA and a 10.8% lower level of 22:5n-6 in RBC (*p* < 0.05; Supplementary Materials, Table S2).

### 3.4. Associations between FADS Genotype and FA Concentrations in Erythrocyte Membranes and Enzyme Activities

The associations between *FADS1* and *FADS2* polymorphisms and FA in RBC are shown in Table 4. The rs174561 polymorphism of *FADS1* was associated with 20:4n-6 concentration in RBC (*p* < 0.05), but there were no other associations between other FAs in RBC levels and the *FADS1* or *FADS2* polymorphisms. Enzyme activities are described in Table 5. We also examined the associations between gene polymorphism and desaturase or elongase activities (Table 6). The D5D value was significantly lower (15.3–17.9%) in subjects with at least one minor allele of any of the examined polymorphisms (*p* < 0.001). In addition, rs174561 and rs3834458 were associated with 22:6n-3/20:5n-3 ratio (*p* < 0.05), and women with at least one minor allele had over 12.0% higher values of this parameter (Table 6). However, the other indices (D6D, 20:4n-6/18:2n-6, 22:4n-6/18:2n-6, 20:5n-3/18:3n-3 and 22:6n-3/18:3n-3) were similar between genotype groups.

We also tested whether and how the dietary intake of protein (as well as enzyme substrates and products) affected enzyme activities. Protein intake affected desaturase activities and the 22:6n-3/20:5n-3 and the 22:6n-3/22:5n-3 ratio was lower in women who met their needs for protein. Specifically, 22:6n-3/20:5n-3: 4.49 ± 0.17 versus 4.01 ± 0.16 (*p* < 0.05) and 22:6n-3/22:5n-3: 1.03 ± 0.07 as against 0.78 ± 0.06 (*p* < 0.05) in women who met and failed to meet the needs for protein, respectively. The same type of association was found for protein intake and lipogenic index (the ratio of 16:0 to 18:2n-6 which reflects *de novo* lipogenesis [39,40]) (1.80 ± 0.07 vs. 0.78 ± 0.06, *p* < 0.05) (data not shown). Moreover, there was no effect of the intake of a product or a substrate of the desaturases on those parameters. Additionally, associations between the examined polymorphisms and the desaturase activities were tested in the regression models. Each of the analyzed polymorphisms were associated with the D5D value, and possessing at least one minor allele was associated with about 25% higher D5D activity (Table 6). Moreover, rs174561 and rs3834458 were associated with the 22:6n-3/20:5n-3 ratio, and women with at least one minor allele had over 15% higher values of this parameter (Table 6).

## 4. Discussion

To the best of our knowledge, this is the first study to investigate the relationship between FA composition in erythrocyte membranes, *FADS1* and *FADS2* gene polymorphism, and macronutrient intake (specifically FA intake) in centrally obese postmenopausal Polish women.

Generally, we saw only a few statistically significant associations between the amounts of protein, fat, and carbohydrates in the diet and the FA profile in erythrocyte membranes (Table 3). We observed that high protein intake and low carbohydrate intake were associated with higher levels of some examined FAs in RBC. There have been no studies of the associations between macronutrient intakes and FA profile, but Novak and Innis (2012) found that protein intake and EPA and DHA intakes were positively correlated, while there was no such correlation with fat intake [41]. For this reason, we anticipated that protein intake might affect the concentrations of EPA, DHA, and other FAs in RBC. Although these particular associations were not observed in our study, the results overall confirm previous suggestions that recommendations for EPA and DHA intake should focus on protein sources, rather than on dietary fat [41,42]. Generally, guidelines for consumers have acknowledged the link between protein sources and suggest weekly numbers of fish servings to provide EPA and DHA [43]. It should be underlined, however, that in populations where seafood intake is low, as in our study, animal-derived foods are important sources of fatty acids. Most likely the observed associations between protein intake and particular fatty acids concentrations (16:1n-7, 18:1n-9c, 18:2n-6 or 18:1n-7c) result from the diet structure characteristic of Poland, where dairy and poultry products are often the main protein sources [44,45]. In our study, around 65% of proteins were derived from products of animal origin. Moreover, pork and poultry provided over 30% of the total protein intake, while milk and dairy products made up more than 20% of the total protein intake. Fish were consumed less frequently and provided around 4% of total protein (data not shown). Interestingly, we also observed that protein intake was associated with the 22:6n-3/20:5n-3 and 22:6n-3/22:5n-3 ratios, and that the values of these indices were lower in women who met their needs for protein (1.2 g of protein/kg of body mass/day). It is also worth mentioning that only concentrations of 18:2n-6t in RBC were associated with the intake of all macronutrients—higher 18:2n-6t levels in RBC were associated with higher total protein, or fat intake, or with lower carbohydrate intake. Trans-FAs are provided by high protein and high-fat products and are formed by technological processes [46]. Together, these results show that dietary habits related to protein intake may affect the FA profile in RBC, and increased fish intake is advisable in Polish postmenopausal women. This is of great importance for this age group, as the adequate intake of food items that are sources of n-3 LC-PUFA can lead to an increase in n-3 LC-PUFA in the RBC, an improvement in plasma lipid profile, and a reduction in cardiovascular risk factors and inflammatory mediators [47].

Furthermore, we examined the relationships between FA intake and composition in erythrocyte membranes. Associations were observed between high SFA intake and high 18:2n-6t, and 17:0 and low 22:4n-6 in RBC (Table S1), and also between high MUFA intake and low 22:5n-6 in RBC. Other studies have described several associations between SFA and unSFA from diet and FA status in the human sample [48,49,50,51,52,53,54,55] and, as mentioned above, FA status may significantly contribute to metabolic health. It has been shown that high levels of total SFA, low levels of PUFA, and high levels of different FAs in human plasma or RBC (mainly, 16:0, 16:1n-7, 18:0, n-6 (18:3n-6, 20:3n-6, AA), and EPA) are associated with the risk of obesity or MetS [56,57,58,59,60]. That we did not find some of the associations often found in other studies might be due to age, sex, postmenopausal status, or central obesity in our study group. The differences could also be explained by the fact that, in our study, we examined FAs in RBC, while the other studies studied FA in serum and plasma (or plasma phospholipids).

The n-3 and n-6 PUFA pathways compete with each other for desaturases and elongases. High levels of dietary precursors (ALA and LA) in human samples could reflect high dietary intake of these FA, or diminished conversion to LC-PUFA. D5D, encoded by *FADS1*, is involved in the conversion of 20:4n-3 to 20:5n-3 and of 20:3n-6 to 20:4n-6 [61,62], and *FADS1* genotype may be associated with metabolic efficiency. In our study, rs174561 polymorphism of the *FADS1* gene was associated with higher 20:4n-6 concentration in RBC in the major allele carriers (*p* < 0.05), but there were no other associations between other FAs in RBC levels and *FADS1* or *FADS2* polymorphisms (Table 4). This is consistent with the results of several previous studies [11,12,13,22,63], where the minor allele carriers of rs174556, rs174547, rs174561, and rs3834458 had higher concentrations of 18:2n-6, 20:3n-6, 18:3n-3 and lower concentrations of 18:3n-6, 20:4n-6, 22:4n-6, 20:5n-3, and 22:5n-3 in serum, plasma phospholipids or RBC. A high level of AA and a low level of EPA, as well as a high AA/EPA ratio, are associated with cardiovascular disorders [64]. AA is a precursor in the conversion of proinflammatory compounds, such as prostaglandins and leukotrienes, so higher levels of AA in the major allele carriers could result in increased inflammation [65,66]. Moreover, in contrast to these studies, in our study, subjects with at least one minor allele of rs174561 had lower levels of 18:2n-6 (Table 4).

In our study, desaturase and elongase activities were estimated using product-to-substrate ratios. The values of D6D obtained were comparable to those observed in other studies with healthy subjects, but the D5D values were higher in our study [52,67]. High D5D activity is associated with lower risk of cardiovascular incidences and also with good insulin sensitivity [52,65] while a low D6D activity index is associated with a lower risk of mortality, insulin resistance, and obesity [52,57,65,68]. The mean D5D and D6D values observed in our postmenopausal centrally obese study sample thus seem surprising, and the reasons for this are unknown. It has been indicated that diet, environmental exposure, and life stage can potentially affect elongase and desaturase activity [1]. Aging may decrease D6D activity in rodents and humans. Decreased activity of D6D with age may not apply to different cohorts, as either males or mixed gender samples have usually been analyzed [69]. Studies of aging women were unable to document a similar decline in enzyme activity, primarily because of the released suppression of desaturase activity with the loss of estrogen after menopause. Estrogen is known to suppress fatty acid desaturation in cell culture and rats [69]. It was also noted that insulin, though not estrogen, appears to influence desaturase activity, as demonstrated by the increased desaturase function associated with hyperinsulinemia in obese women [70]. It should, however, be underlined that desaturase indices, calculated as product-to-substrate ratios, are only an indirect measure of desaturase activity.

We additionally observed that the mean D5D value was significantly lower in the minor allele carriers of rs174556, rs174561, and rs174547 of *FADS1* and of rs3834458 of the *FADS2* gene. There have been only a few studies to examine the associations between *FADS* genotype and desaturase and elongase activity in women but, to the best of our knowledge, there has been no study on this topic in postmenopausal women; this limits interpretation [71,72,73,74]. However, our results are consistent with previous findings of decreased desaturase activities in minor allele carriers [23].

As shown in our study and several previous studies, FA metabolism depends on diet and genetically determined desaturase activities. These studies have practical implications: In particular, people with unfavorable genotypes and higher intake of n-3 FAs—mainly of 18:3n-3, DHA, and EPA—might be advised to reduce the risk of inflammation or MetS.

One strength of the presented study is that FA composition was analyzed in erythrocyte membranes, which reflects the long-term FA intake and it is known as the best noninvasive biomarker of FA intake in humans. Moreover, the study group was well defined and homogenous, leading to low interindividual variability. One weakness of this study is the small sample size. Moreover, the product-to-substrate estimation, a traditional approach, was used to assess enzymatic activity, but no protein activity was determined. Another limitation could be that we did not include all the potential confounders in the multiple regression analysis. These include, for example, data on liver functioning. Further, to assess habitual intake of FAs we did not use a retrospective method. It is however worth mentioning that our study presents for the first time the relationship between macronutrients and FA intake and genotype and FA composition in erythrocyte membranes in postmenopausal Polish women.

## 5. Conclusions

We conclude that protein or carbohydrate intake may be associated with FA concentration in the RBC in centrally obese postmenopausal Polish women. Moreover, SFA, MUFA, or PUFA may not be associated with FA levels in RBC in this study group. The potential implications of these findings should be investigated in a further intervention study considering non obese population. The D5D value, though not the D6D value, may be affected by *FADS1* or *FADS2* polymorphism.

## Figures and Tables

**Table 1:** Primer and probe sequences used in the *FADS* genotyping assay.

Gene	SNP	Primers	Probes
*FADS1*	rs174556 (C > T)	5’ACAAGGGCCTTGTGAAGAAGT 5’GCCTGTGACCTCATGACTATGC	5’GAGTCTAGATGGaATCACAGTCATAGT--FL
	rs174561 (T > C)	5’GCACCACACATACGGACCAAT 5’GGGTCAACCAGAGTGACCACTC	5’ GCATCCCCGGCCCCA--FL
	rs174547 (T > C)	5’TGGGTGACACAGATGAACCATATTC 5’GGCTAATGAGAAAATGCTGTTTGG	5’CTACGCACCCTTTTCAATAGTTG--FL
*FADS2*	rs3834458 (delT)	5’TTACTGAGACCAGGGCAAGGAC 5’CGGCAGTCGAGACTCCAGTATC	5’TCAGACAATCTT_GAAAAGAATTGC

*FADS*: Fatty acid desaturase; SNP: Single-nucleotide polymorphism; *FADS1*: Fatty acid desaturase 1; *FADS2*: Fatty acid desaturase 2.

**Table 2:** Dietary macronutrient and fatty acid intakes among postmenopausal women.

Macronutrient.	Daily Intake					
	Mean ± SEM	Median	Quartile 1	Quartile 2	Quartile 3	Quartile 4
Total protein (g)	67.6 ± 1.6	66.5	45.5 ± 1.2	61.2 ± 0.6	71.0 ± 0.5	93.3 ± 2.6
Total carbohydrates (g) ^a^	255.7 ± 11.1	229.5	128.6 ± 3.8	191.6 ± 3.3	265.6 ± 3.9	437.2 ± 20.5
Total lipids (g)	52.4 ± 2.0	47.4	27.4 ± 0.8	41.0 ± 0.7	57.4 ± 0.9	83.8 ± 3.6
Total SFAs (g)	20.1 ± 0.9	18.4	9.9 ± 0.3	15.2 ± 0.3	21.7 ± 0.3	33.7 ± 1.8
4:0 (mg)	315.4 ± 26.7	258.3	90.7 ± 6.3	201.1 ± 4.8	315.8 ± 5.6	654.2 ± 79.3
6:0 (mg)	211.6 ± 16.8	171.6	71.7 ± 4.4	135.0 ± 2.9	210.8 ± 3.7	428.7 ± 49.6
8:0 (mg)	153.2 ± 10.7	129.9	56.7 ± 3.0	104.5 ± 2.4	156.4 ± 2.7	295.3 ± 30.2
10:0 (mg)	378.6 ± 26.1	318.6	136.0 ± 6.4	258.0 ± 5.9	379.9 ± 6.6	740.5 ± 70.7
12:0 (mg)	558.3 ± 35.6	468.3	209.5 ± 10.6	379.0 ± 8.1	578.4 ± 8.8	1067.0 ± 91.6
14:0 (g)	2.2 ± 0.1	1.9	0.9 ± 0.03	1.6 ± 0.03	2.2 ± 0.04	3.9 ± 0.3
15:0 (mg)	252.4 ± 14.4	215.9	95.1 ± 5.0	178.6 ± 3.9	264.4 ± 5.0	471.9 ± 32.0
16:0 (g)	11.2 ± 0.5	10.3	5.7 ± 0.2	8.7 ± 0.2	12.1 ± 0.2	18.3 ± 0.8
17:0 (mg)	173.8 ± 10.6	151.5	66.7 ± 3.4	121.0 ± 3.0	179.5 ± 2.9	328.0 ± 26.4
18:0 (g)	4.6 ± 0.2	4.1	2.0 ± 0.1	3.3 ± 0.1	5.0 ± 0.1	8.1 ± 0.4
20:0 (mg)	75.7 ± 4.8	62.9	20.0 ± 1.2	47.9 ± 1.2	81.3 ± 2.0	153.9 ± 9.0
Total MUFA (g)	20.4 ± 08	19.2	9.7 ± 0.3	15.8 ± 0.4	22.3 ± 0.4	33.7 ± 1.3
14:1 (mg)	193.0 ± 13.3	164.7	74.0 ± 3.8	132.7 ± 2.8	196.5 ± 2.9	370.4 ± 37.4
15:1 (mg)	67.1 ± 5.9	48.3	21.8 ± 1.4	40.6 ± 0.9	65.4 ± 1.6	140.8 ± 18.0
16:1 (g)	1.2 ± 0.1	1.1	0.5 ± 0.03	1.0 ± 0.02	1.4 ± 0.02	2.1 ± 0.1
17:1 (mg)	111.6 ± 9.2	90.1	36.6 ± 2.1	69.7 ± 1.8	111.1 ± 1.9	228.7 ± 27.3
18:1 (g)	18.3 ± 0.8	16.8	8.7 ± 0.3	14.1 ± 0.3	19.9 ± 0.3	30.6 ± 1.1
20:1 (mg)	239.1 ± 15.9	191.3	73.6 ± 4.8	155.0 ± 3.0	239.5 ± 6.2	488.5 ± 35.2
22:1 (mg)	156.2 ± 21.3	51.8	1.1 ± 0.3	23.7 ± 2.3	115.6 ± 8.1	483.2 ± 54.2
PUFA total (g)	7.4 ± 0.4	6.1	3.8 ± 0.1	5.5 ± 0.1	7.3 ± 0.1	13.0 ± 1.1
18:2 (g)	5.8 ± 0.3	4.9	3.0 ± 0.1	4.5 ± 0.1	5.8 ± 0.1	9.8 ± 0.5
18:3 (g)	1.3 ± 0.2	0.8	0.5 ± 0.01	0.7 ± 0.01	1.0 ± 0.02	3.1 ± 0.7
18:4 (mg)	9.7 ± 2.2	0.0	0.0 ± 0.0	0.0 ± 0.0	2.8 ± 0.3	37.5 ± 6.9
20:3 (mg)	0.6 ± 0.3	0.0	0.0 ± 0.0	0.0 ± 0.0	0.0 ± 0.0	9.4 ± 4.4
20:4 (mg)	119.5 ± 8.7	91.0	30.7 ± 2.3	68.9 ± 2.0	123.1 ± 4.1	255.2 ± 19.1
20:5 (mg)	59.3 ± 10.4	11.3	0.2 ± 0.1	5.6 ± 0.4	27.0 ± 2.1	203.7 ± 30.3
22:5 (mg)	23.8 ± 3.9	5.9	0.1 ± 0.1	3.7 ± 0.2	11.4 ± 0.5	79.8 ± 10.8
22:6 (mg)	139.4 ± 21.6	50.3	9.6 ± 1.0	34.6 ± 1.4	82.4 ± 3.5	429.4 ± 64.8

^a^ Calculations of total carbohydrate intake did not include dietary fiber and resistant starch. SFA: Saturated fatty acids; MUFA: Monounsaturated fatty acids; PUFA: Polyunsaturated fatty acids; SEM: standard error of mean; *N* = 143.

**Table 3:** Concentrations of erythrocyte fatty acids stratified by macronutrient intake.

Concentrations of FA in RBC (µg/mL)	Low Protein ^a^	High Protein ^a^	*p*-Value	Low Carbohydrates ^a^	High Carbohydrates ^a^	*p*-Value	Low Fat ^a^	High Fat ^a^	*p*-Value
14:0	10.23 ± 9.48	13.39 ± 12.88	0.213	12.01 ± 11.49	11.58 ± 11.31	0.924	11.28 ± 10.86	12.32 ± 11.91	0.812
15:0	12.08 ± 5.66	12.15 ± 5.03	0.893	12.20 ± 4.81	12.02 ± 5.86	0.976	12.35 ± 5.77	11.88 ± 4.89	0.425
16:0	417.76 ± 148.18	456.41 ± 189.46	0.316	440.62 ± 175.05	433.19 ± 166.75	0.902	426.90 ± 157.45	447.13 ± 183.22	0.694
17:0	13.01 ± 10.82	15.66 ± 9.53	0.185	15.41 ± 9.48	13.23 ± 10.94	0.330	13.65 ± 10.66	15.01 ± 9.84	0.637
18:0	273.15 ± 55.14	275.71 ± 62.72	0.973	273.11 ± 59.65	275.75 ± 58.37	0.640	272.05 ± 58.44	276.83 ± 59.53	0.896
22:0	6.44 ± 3.71	6.45 ± 3.51	0.877	6.32 ± 3.59	6.57 ± 3.63	0.661	6.46 ± 3.49	6.42 ± 3.73	0.861
14:1n-9	4.58 ± 2.71	5.29 ± 2.59	0.254	5.02 ± 2.50	4.84 ± 2.84	0.909	4.94 ± 2.91	4.92 ± 2.41	0.762
14:1n-5	2.81 ± 2.68	2.32 ± 1.72	0.120	2.73 ± 2.52	2.40 ± 1.96	0.533	2.34 ± 1.97	2.79 ± 2.51	0.420
15:1	27.66 ± 11.10	27.78 ± 8.94	0.895	26.74 ± 8.24	28.71 ± 11.58	0.213	27.95 ± 11.04	27.48 ± 9.02	0.887
16:1n-7	17.47 ± 18.29	27.17 ± 26.81	0.035	24.16 ± 25.01	20.37 ± 21.54	0.424	20.03 ± 20.65	24.57 ± 25.74	0.387
17:1	34.47 ± 19.87	32.03 ± 16.66	0.567	29.61 ± 14.93	36.97 ± 20.68	0.020	33.97 ± 19.49	32.54 ± 17.17	0.914
18:1n-9t	2.64 ± 1.80	2.67 ± 1.73	0.959	2.56 ± 1.73	2.75 ± 1.79	0.493	2.65 ± 1.69	2.66 ± 1.84	0.898
18:1n-7t	4.79 ± 3.14	4.42 ± 2.40	0.448	4.62 ± 2.73	4.59 ± 2.88	0.950	4.53 ± 3.07	4.68 ± 2.50	0.799
18:1n-9c	354.50 ± 162.53	403.01 ± 209.02	0.176	383.59 ± 202.80	373.47 ± 172.90	0.744	369.89 ± 166.72	387.38 ± 208.17	0.665
18:1n-7c	27.54 ± 10.52	33.46 ± 15.87	0.022	31.53 ± 14.88	29.40 ± 12.45	0.457	29.27 ± 12.79	31.70 ± 14.60	0.403
18:1n-5c	3.41 ± 2.42	5.26 ± 4.28	0.007	4.90 ± 4.12	3.75 ± 2.85	0.112	3.95 ± 3.20	4.72 ± 3.91	0.338
20:1n-9	7.09 ± 2.88	7.32 ± 2.63	0.621	7.33 ± 2.80	7.07 ± 2.72	0.674	7.26 ± 2.71	7.15 ± 2.81	0.723
22:1n-9	12.36 ± 8.93	10.98 ± 8.03	0.426	10.52 ± 7.38	12.86 ± 9.39	0.139	12.15 ± 9.03	11.20 ± 7.94	0.765
24:1n-9	13.89 ± 12.59	16.34 ± 11.06	0.237	15.34 ± 10.97	14.88 ± 12.81	0.946	14.09 ± 12.33	16.14 ± 11.40	0.312
18:2n-6t	7.59 ± 4.36	10.47 ± 4.55	0.001	9.89 ± 4.62	8.14 ± 4.58	0.051	7.99 ± 4.53	10.07 ± 4.60	0.018
18:2n-6	263.82 ± 161.35	326.61 ± 217.22	0.092	312.01 ± 207.61	277.65 ± 176.79	0.333	276.75 ± 175.44	313.46 ± 209.02	0.381
18:3n-6	4.75 ± 3.71	6.45 ± 5.19	0.052	6.07 ± 5.05	5.11 ± 4.00	0.289	5.06 ± 3.98	6.13 ± 5.07	0.216
20:2n-6	4.69 ± 8.70	5.25 ± 8.89	0.641	5.29 ± 8.85	4.65 ± 8.74	0.634	5.67 ± 10.16	4.27 ± 7.09	0.405
20:3n-6	30.84 ± 9.37	33.49 ± 12.71	0.316	31.94 ± 11.10	32.37 ± 11.37	0.702	32.12 ± 11.44	32.19 ± 11.02	0.783
20:4n-6	278.58 ± 55.85	283.64 ± 58.84	0.939	282.06 ± 59.92	280.10 ± 54.72	0.804	280.99 ± 57.55	281.19 ± 57.27	0.534
22:2n-6	2.91 ± 1.54	3.00 ± 1.71	0.787	3.05 ± 1.71	2.86 ± 1.53	0.434	2.91 ± 1.66	3.00 ± 1.60	0.729
22:4n-6	47.91 ± 12.16	44.41 ± 11.58	0.068	44.45 ± 11.80	47.92 ± 11.96	0.060	48.33 ± 12.03	43.98 ± 11.57	0.019
22:5n-6	10.69 ± 3.52	10.81 ± 3.34	0.907	10.27 ± 3.46	11.24 ± 3.33	0.070	11.07 ± 3.45	10.44 ± 3.39	0.223
18:3n-3t	2.33 ± 2.33	2.61 ± 2.95	0.672	2.41 ± 2.35	2.54 ± 2.94	0.695	2.16 ± 2.24	2.79 ± 2.99	0.177
18:3n-3	13.17 ± 9.30	14.16 ± 8.18	0.578	13.46 ± 8.31	13.86 ± 9.21	0.589	13.17 ± 8.96	14.15 ± 8.55	0.640
20:3n-3	9.06 ± 8.32	12.08 ± 9.53	0.072	11.46 ± 9.71	9.63 ± 8.26	0.353	9.95 ± 8.61	11.16 ± 9.48	0.539
20:5n-3	25.45 ± 11.14	27.74 ± 14.05	0.320	27.58 ± 14.08	25.57 ± 11.09	0.467	24.66 ± 9.54	28.54 ± 15.04	0.123
22:5n-3	134.26 ± 61.66	136.29 ± 45.00	0.990	142.04 ± 50.02	128.38 ± 57.03	0.193	133.53 ± 53.37	137.03 ± 54.68	0.986
22:6n-3	104.81 ± 29.13	97.72 ± 26.87	0.164	99.78 ± 25.44	102.83 ± 30.79	0.477	100.25 ± 29.90	102.35 ± 26.44	0.745
20:3n-9	5.50 ± 2.18	5.65 ± 2.72	0.682	5.55 ± 2.69	5.60 ± 2.21	0.928	5.38 ± 2.03	5.77 ± 2.83	0.254

Values are means ± standard deviations (SDs) for fatty acids levels. ^a^ Low and high intakes determined by medians for proteins, carbohydrates, and fat, which were 16.85% energy intake, 54.86% energy intake, and 27.05% energy intake, respectively. The model was adjusted for body mass index (BMI), PA, and hypolipidemic and hypoglycemic medications. Abbreviations: SFA: saturated fatty acids; MUFA: monounsaturated fatty acids; n-3 PUFA: n-3 polyunsaturated fatty acids; n-6 PUFA: n-6 polyunsaturated fatty acids; RBC: red blood cells; BMI: body mass index; PA: physical activity.

**Table 4:** Associations between *FADS1* and *FADS2* polymorphisms and fatty acid concentrations in erythrocyte membranes.

Variable	Polymorphisms											
	rs174556 *FADS1*			rs174547 *FADS1*			rs174561 *FADS1*			rs3834458 *FADS2*		
	CT + TT *N* = 53	CC *N* = 72	*p*-Value	TC + CC *N* = 54	TT *N* = 72	*p*-Value	TC + CC *N* = 58	TT *N* = 67	*p*-Value	T/- + -/- *N* = 59	TT *N* = 66	*p*-Value
n-6 PUFA in RBC (µg/mL)												
18:2n-6t	9.19 ± 4.54	8.85 ± 4.84	NS	9.24 ± 4.51	8.85 ± 4.84	NS	8.90 ± 4.38	9.08 ± 4.99	NS	8.90 ± 4.58	9.08 ± 4.83	NS
18:2n-6	312.16 ± 202.00	286.07 ± 188.05	NS	309.59 ± 200.98	286.07 ± 188.05	NS	293.42 ± 192.49	300.35 ± 196.16	NS	304.90 ± 193.68	290.19 ± 194.97	NS
18:3n-6	5.60 ± 3.80	5.67 ± 5.12	NS	5.54 ± 3.79	5.67 ± 5.12	NS	5.55 ± 3.77	5.72 ± 5.22	NS	5.61 ± 3.87	5.67 ± 5.18	NS
20:2n-6	4.40 ± 6.85	5.53 ± 10.04	NS	4.32 ± 6.81	5.53 ± 10.04	NS	4.39 ± 6.61	5.62 ± 10.37	NS	4.19 ± 6.54	5.81 ± 10.44	NS
20:3n-6	34.06 ± 11.04	30.81 ± 11.32	NS	34.05 ± 10.94	30.81 ± 11.32	NS	32.97 ± 10.85	31.50 ± 11.66	NS	33.63 ± 11.06	30.90 ± 11.39	NS
20:4n-6	267.92 ± 55.96	289.97 ± 57.23	NS	268.81 ± 55.81	289.97 ± 57.23	NS	265.27 ± 54.84	293.90 ± 56.85	<0.05	270.18 ± 54.81	289.95 ± 58.67	NS
22:2n-6	2.96 ± 1.87	2.96 ± 1.45	NS	2.96 ± 1.86	2.96 ± 1.45	NS	2.90 ± 1.80	3.02 ± 1.49	NS	3.02 ± 1.91	2.91 ± 1.36	NS
22:4n-6	44.97 ± 13.65	47.10 ± 10.70	NS	45.07 ± 13.54	47.10 ± 10.70	NS	45.01 ± 12.96	47.22 ± 11.17	NS	45.35 ± 13.67	46.95 ± 10.41	NS
22:5n-6	11.06 ± 3.67	10.57 ± 3.27	NS	11.03 ± 3.65	10.57 ± 3.27	NS	10.91 ± 3.41	10.67 ± 3.49	NS	11.01 ± 3.57	10.58 ± 3.34	NS
n-3 PUFA in RBC (µg/mL)												
18:3n-3t	2.22 ± 1.93	2.70 ± 3.09	NS	2.20 ± 1.92	2.70 ± 3.09	NS	2.13 ± 1.44	2.82 ± 3.37	NS	2.23 ± 1.89	2.74 ± 3.20	NS
18:3n-3	12.96 ± 7.10	14.33 ± 9.84	NS	12.84 ± 7.09	14.33 ± 9.84	NS	12.40 ± 6.65	14.92 ± 10.18	NS	12.72 ± 7.17	14.67 ± 9.97	NS
20:3n-3	10.62 ± 8.29	10.74 ± 9.62	NS	10.47 ± 8.28	10.74 ± 9.62	NS	10.03 ± 7.73	11.26 ± 10.07	NS	11.02 ± 9.30	10.40 ± 8.87	NS
20:5n-3	24.89 ± 12.80	27.97 ± 12.64	NS	24.75 ± 12.72	27.97 ± 12.64	NS	24.42 ± 12.79	28.61 ± 12.48	NS	24.73 ± 12.56	28.40 ± 12.76	NS
22:5n-3	129.50 ± 54.13	138.00 ± 53.68	NS	130.53 ± 54.16	138.00 ± 53.68	NS	128.93 ± 54.39	139.13 ± 53.27	NS	132.32 ± 53.54	136.26 ± 54.40	NS
22:6n-3	101.03 ± 29.62	101.13 ± 27.15	NS	100.76 ± 29.40	101.13 ± 27.15	NS	100.21 ± 29.35	101.85 ± 27.18	NS	101.01 ± 28.77	101.16 ± 27.72	NS

Values are means ± SDs for fatty acids levels. The model was adjusted for BMI, PA, and hypolipidemic and hypoglycemic medications. Abbreviations: SFA: saturated fatty acids; MUFA: monounsaturated fatty acids; n-3 PUFA: n-3 polyunsaturated fatty acids; n-6 PUFA: n-6 polyunsaturated fatty acids; RBC: Red blood cells; BMI: Body mass index; PA: Physical activity, NS: not significant.

**Table 5:** Desaturase and elongase index characteristics.

Parameter	Mean ± SD	Minimum	Maximum
Desaturase activity ratios			
D5D	9.40 ± 0.21	4.95	15.37
D6D	0.021 ± 0.001	0.003	0.057
Combined effect of desaturase and elongase activity ratios			
20:4n-6/18:2n-6	1.24 ± 0.05	0.32	2.34
22:6n-3/20:5n-3	4.27 ± 0.12	1.22	8.98
20:5n-3/18:3n-3	2.41 ± 0.12	0.48	8.40
22:6n-3/18:3n-3	10.16 ± 0.52	2.12	27.38
22:4n-6/18:2n-6	0.22 ± 0.01	0.03	0.50

*N* = 130; abbreviations: D5D: Delta-5 desaturase index, 20:4n-6/20:3n-6; D6D: delta-6 desaturase index, 18:3n-6/18:2n-6.

**Table 6:** Associations between *FADS1* and *FADS2* polymorphisms and desaturase and elongase indexes.

Variable	Polymorphisms											
	rs174556 *FADS1*			rs174547 *FADS1*			rs174561 *FADS1*			rs3834458 *FADS2*		
	CT + TT *N* = 53	CC *N* = 72	*p*-Value	TC + CC *N* = 54	TT *N* = 72	*p*-Value	TC + CCN *N* = 58	TT *N* = 67	*p*-Value	T/- + -/- *N* = 59	TT *N*= 66	*p*-Value
Desaturase activity ratios												
D5D	8.29 ± 1.97	10.10 ± 2.37	<0.0001	8.31 ± 1.96	10.10 ± 2.37	<0.0001	8.51 ± 2.12	10.04 ± 2.37	< 0.001	8.52 ± 2.14	10.06 ± 2.36	<0.001
D6D	0.02 ± 0.01	0.02 ± 0.01	NS	0.02 ± 0.01	0.02 ± 0.01	NS	0.02 ± 0.01	0.02 ± 0.01	NS	0.02 ± 0.01	0.02 ± 0.01	NS
Combined effect of desaturase and elongase activity ratios												
20:4n-6/18:2n-6	1.14 ± 0.52	1.30 ± 0.50	<0.05	1.15 ± 0.52	1.30 ± 0.50	NS	1.18 ± 0.50	1.27 ± 0.52	NS	1.16 ± 0.50	1.30 ± 0.51	NS
22:4n-6/18:2n-6	0.21 ± 0.12	0.23 ± 0.12	NS	0.21 ± 0.12	0.23 ± 0.12	NS	0.22 ± 0.12	0.22 ± 0.12	NS	0.21 ± 0.12	0.23 ± 0.12	NS
20:5n-3/18:3n-3	2.29 ± 1.28	2.52 ± 1.43	NS	2.29 ± 1.27	2.52 ± 1.43	NS	2.30 ± 1.25	2.52 ± 1.47	NS	2.33 ± 1.26	2.50 ± 1.47	NS
22:6n-3/18:3n-3	10.01 ± 5.61	10.22 ± 6.39	NS	10.06 ± 5.57	10.22 ± 6.39	NS	10.33 ± 5.80	9.95 ± 6.29	NS	10.37 ± 5.84	9.91 ± 6.26	NS
22:6n-3/20:5n-3	4.49 ± 1.34	4.04 ± 1.28	NS	4.50 ± 1.33	4.04 ± 1.28	NS	4.57 ± 1.36	3.95 ± 1.23	<0.05	4.53 ± 1.36	3.97 ± 1.24	<0.05

Values are means ± standard deviations (SDs) for fatty acids levels. The model was adjusted for age, body mass, physical activity, hypolipidemic and hypoglycemic medications, and intake of enzyme precursors. Abbreviations: D5D: delta-5 desaturase index, 20:4n-6/20:3n-6; D6D: delta-6 desaturase index, 18:3n-6/18:2n-6.

## Supplementary Materials

The following are available online at http://www.mdpi.com/2072-6643/10/8/1068/s1, Table S1: Distribution of erythrocyte fatty acids by median total SFA intake, Table S2: Distribution of erythrocyte fatty acids by median total MUFA intake, Table S3: Distribution of erythrocyte fatty acids by median total PUFA intake.
