# Supplementary material for: Associations between Fatty Acid Intake and Status, Desaturase Activities, and FADS Gene Polymorphism in Centrally Obese Postmenopausal Polish Women
Source: Nutrients. 2018 Aug 10;10(8):1068. doi: 10.3390/nu10081068 (PMC6115977; doi:10.3390/nu10081068)
Supplement: Supplementary file 1 [file nutrients-10-01068-s001.pdf]

Supplementary Materials: Associations Between Fatty Acid Intake and Status, Desaturase Activities, and *FADS* Gene Polymorphism in Centrally Obese Postmenopausal Polish Women

Agata Muzsik [1], Joanna Bajerska [1], Henryk H. Jeleń [2], Anna Gaca [2] and Agata Chmurzynska [1,*]

Table S1. Distribution of erythrocyte fatty acids by median total SFA intake.

Concentrations of FA in RBC (μg/mL)	Low SFA Group [a]	High SFA Group [a]	*p*-Value
14:0	11.01 ± 10.66	12.63 ± 12.09	0.348
15:0	11.88 ± 5.40	12.36 ± 5.31	0.630
16:0	422.73 ± 158.05	451.82 ± 182.46	0.276
17:0	12.55 ± 7.58	16.20 ± 12.24	0.034
18:0	272.65 ± 58.58	276.28 ± 59.46	0.729
22:0	6.64 ± 3.82	6.24 ± 3.37	0.401
14:1*n-9*	4.62 ± 2.48	5.26 ± 2.84	0.215
14:1*n-5*	2.40 ± 1.96	2.75 ± 2.54	0.377
15:1	28.76 ± 11.01	26.63 ± 8.89	0.156
16:1*n-7*	19.21 ± 20.17	25.51 ± 26.03	0.094
17:1	35.69 ± 19.68	30.72 ± 16.56	0.078
18:1*n-9t*	2.65 ± 1.75	2.67 ± 1.79	0.993
18:1*n-7t*	4.42 ± 3.05	4.81 ± 2.52	0.433
18:1*n-9c*	359.67 ± 162.82	398.38 ± 210.52	0.172
18:1*n-7c*	28.75 ± 12.64	32.28 ± 14.65	0.127
18:1*n-5c*	3.91 ± 3.38	4.78 ± 3.76	0.195
20:1*n-9*	7.31 ± 2.98	7.10 ± 2.51	0.628
22:1*n-9*	12.48 ± 9.85	10.85 ± 6.76	0.303
24:1*n-9*	14.93 ± 12.83	15.30 ± 10.89	0.982
18:2*n-6t*	8.15 ± 4.59	9.93 ± 4.61	0.028
18:2*n-6*	266.97 ± 167.56	324.32 ± 213.85	0.070
18:3*n-6*	4.99 ± 3.92	6.23 ± 5.12	0.104
20:2*n-6*	5.86 ± 10.23	4.04 ± 6.87	0.260
20:3*n-6*	31.21 ± 11.34	33.15 ± 11.04	0.297
20:4*n-6*	280.97 ± 57.36	281.22 ± 57.46	0.895
22:2*n-6*	2.87 ± 1.63	3.05 ± 1.63	0.456
22:4*n-6*	48.06 ± 11.86	44.19 ± 11.83	0.045
22:5*n-6*	11.10 ± 3.70	10.40 ± 3.09	0.163
18:3*n-3t*	2.49 ± 2.82	2.46 ± 2.49	0.921
18:3*n-3*	13.11 ± 8.80	14.23 ± 8.71	0.535
20:3*n-3*	10.21 ± 9.00	10.92 ± 9.13	0.726
20:5*n-3*	26.08 ± 12.90	27.11 ± 12.52	0.693
22:5*n-3*	130.29 ± 54.96	140.49 ± 52.57	0.252
22:6*n-3*	103.33 ± 30.89	99.17 ± 25.03	0.359
20:3*n-9*	5.60 ± 2.69	5.55 ± 2.21	0.976

Values are means ± SD for fatty acids levels. [a]Low and high intakes determined by median SFA, which was 10.48% of energy intake. The model was adjusted for BMI, physical activity, and hypolipidemic and hypoglycemic medications. Abbreviations: SFA: saturated fatty acids; MUFA: monounsaturated fatty acids; *n-3* PUFA: *n-3* polyunsaturated fatty acids; *n-6* PUFA: *n-6* polyunsaturated fatty acids; RBC: red blood cells.

Table S2. Distribution of erythrocyte fatty acids by median of total MUFA inta**ke.**

Concentrations of FA in RBC (μg/mL)	Low MUFA [a]	High MUFA [a]	*p*-Value
14:0	12.07 ± 11.43	11.52 ± 11.38	0.586
15:0	12.33 ± 5.77	11.89 ± 4.88	0.441
16:0	439.38 ± 168.35	434.37 ± 173.75	0.631
17:0	14.01 ± 10.78	14.66 ± 9.74	0.965
18:0	275.78 ± 59.98	272.99 ± 58.00	0.500
22:0	6.85 ± 3.55	6.02 ± 3.62	0.256
14:1*n-9*	5.16 ± 2.91	4.70 ± 2.39	0.240
14:1*n-5*	2.35 ± 1.97	2.80 ± 2.52	0.450
15:1	28.19 ± 11.20	27.22 ± 8.74	0.619
16:1*n-7*	21.83 ± 22.11	22.76 ± 24.73	0.966
17:1	34.73 ± 19.92	31.73 ± 16.50	0.484
18:1*n-9t*	2.71 ± 1.68	2.60 ± 1.85	0.722
18:1*n-7t*	4.45 ± 2.82	4.77 ± 2.79	0.635
18:1*n-9c*	383.63 ± 180.86	373.26 ± 196.32	0.644
18:1*n-7c*	30.19 ± 13.46	30.77 ± 14.08	1.000
18:1*n-5c*	4.28 ± 3.47	4.39 ± 3.72	0.938
20:1*n-9*	7.35 ± 2.79	7.06 ± 2.72	0.403
22:1*n-9*	12.44 ± 8.92	10.89 ± 8.01	0.454
24:1*n-9*	15.45 ± 12.97	14.76 ± 10.71	0.741
18:2*n-6t*	8.58 ± 4.71	9.49 ± 4.62	0.357
18:2*n-6*	291.40 ± 191.76	298.71 ± 195.68	0.927
18:3*n-6*	5.29 ± 4.11	5.91 ± 5.02	0.478
20:2*n-6*	4.73 ± 8.70	5.23 ± 8.90	0.718
20:3*n-6*	32.89 ± 11.53	31.39 ± 10.86	0.255
20:4*n-6*	281.41 ± 55.17	280.76 ± 59.67	0.486
22:2*n-6*	3.03 ± 1.79	2.88 ± 1.44	0.590
22:4*n-6*	47.60 ± 12.11	44.67 ± 11.72	0.098
22:5*n-6*	11.36 ± 3.37	10.13 ± 3.38	0.014
18:3*n-3t*	2.48 ± 2.89	2.47 ± 2.40	0.917
18:3*n-3*	13.82 ± 9.04	13.49 ± 8.49	0.600
20:3*n-3*	10.94 ± 9.31	10.16 ± 8.80	0.529
20:5*n-3*	25.72 ± 11.15	27.49 ± 14.14	0.635
22:5*n-3*	134.01 ± 55.10	136.59 ± 52.90	0.957
22:6*n-3*	100.69 ± 29.53	101.93 ± 26.85	0.945
20:3*n-9*	5.45 ± 2.01	5.71 ± 2.86	0.499

Values are means ± SDs for fatty acids levels. [a]Low and high intakes determined by median MUFA, which was 10.74% of energy intake. Median total energy from MUFA intake per day was 10.74%. The model was adjusted for BMI, physical activity, and hypolipidemic and hypoglycemic medications. Abbreviations: SFA: saturated fatty acids; MUFA: monounsaturated fatty acids; *n-3* PUFA: *n-3* polyunsaturated fatty acids; *n-6* PUFA: *n-6* polyunsaturated fatty acids; RBC: red blood cells.

Table S3. Distribution of erythrocyte fatty acids by median total PUFA intake.

Concentrations of FA in RBC (µg/mL)	Low PUFA [a]	High PUFA [a]	*p*-Value
14:0	10.98 ± 10.25	12.61 ± 12.39	0.461
15:0	12.12 ± 5.62	12.12 ± 5.09	0.939
16:0	428.03 ± 157.50	445.70 ± 182.94	0.602
17:0	13.68 ± 10.89	14.96 ± 9.61	0.468
18:0	271.92 ± 58.20	276.88 ± 59.74	0.702
22:0	6.40 ± 3.38	6.49 ± 3.83	0.878
14:1n-9	4.93 ± 2.82	4.93 ± 2.53	0.875
14:1n-5	2.67 ± 2.23	2.47 ± 2.31	0.548
15:1	27.28 ± 10.98	28.15 ± 9.11	0.638
16:1n-7	20.23 ± 20.31	24.31 ± 25.98	0.346
17:1	32.97 ± 18.42	33.55 ± 18.37	0.840
18:1n-9t	2.52 ± 1.66	2.79 ± 1.86	0.410
18:1n-7t	4.45 ± 2.85	4.76 ± 2.76	0.544
18:1n-9c	370.19 ± 176.51	386.81 ± 199.50	0.604
18:1n-7c	28.80 ± 12.27	32.12 ± 14.91	0.179
18:1n-5c	4.16 ± 3.55	4.50 ± 3.63	0.672
20:1n-9	7.05 ± 2.62	7.36 ± 2.89	0.516
22:1n-9	12.10 ± 9.39	11.27 ± 7.55	0.644
24:1n-9	15.24 ± 13.28	14.98 ± 10.42	0.877
18:2n-6t	8.28 ± 4.56	9.75 ± 4.70	0.092
18:2n-6	279.67 ± 185.88	310.02 ± 199.97	0.400
18:3n-6	5.21 ± 4.09	5.97 ± 5.00	0.353
20:2n-6	4.68 ± 8.72	5.26 ± 8.87	0.683
20:3n-6	32.11 ± 11.50	32.20 ± 10.98	0.925
20:4n-6	275.82 ± 56.41	286.28 ± 57.91	0.378
22:2n-6	2.93 ± 1.67	2.98 ± 1.59	0.871
22:4n-6	47.61 ± 12.75	44.76 ± 11.05	0.151
22:5n-6	10.92 ± 3.24	10.60 ± 3.61	0.511
18:3n-3t	2.16 ± 1.46	2.79 ± 3.43	0.193
18:3n-3	12.45 ± 7.43	14.85 ± 9.78	0.123
20:3n-3	10.68 ± 9.54	10.43 ± 8.58	0.862
20:5n-3	24.59 ± 8.87	28.55 ± 15.36	0.079
22:5n-3	135.33 ± 56.22	135.21 ± 51.84	0.949
22:6n-3	100.04 ± 28.05	102.53 ± 28.41	0.630
20:3n-9	5.46 ± 2.19	5.69 ± 2.70	0.530

Values are means ± SD for fatty acids levels. [a]Low and high intakes determined by median PUFA, which was 3.66% of energy intake. The model was adjusted for BMI, physical activity, and hypolipidemic and hypoglycemic medications. Abbreviations: SFA: saturated fatty acids; MUFA: monounsaturated fatty acids; *n-3* PUFA: *n-3* polyunsaturated fatty acids; *n-6* PUFA: *n-6* polyunsaturated fatty acids; RBC: red blood cells.
